# Rabies risk and response in tea-estate health systems in Assam, India: a constructivist grounded theory analysis

**DOI:** 10.1136/bmjph-2025-004805

**Published:** 2026-09-30

**Authors:** Dipika Bumb, Shanti Priya Kindo, Rasika G Shirke, Aina U Kurup, Chalasani Satwik, Parimala Mohanty, Jully Gogoi-Tiwari, Ganesh Daimary, Riya Shigwan, Harish Kumar Tiwari

**Affiliations:** 1 Ramaiah International Centre for Public Health Innovations, Bangalore, India; 2 Jyoti and Bhupat Mehta School of Health Science and Technology, Indian Institute of Technology Guwahati, Guwahati, Assam, India; 3 DY Patil University School of Public Health, Mumbai, India; 4 Indian Institute of Public Health-Delhi, New Delhi, India; 5 Indian Institute of Technology Guwahati, Guwahati, Assam, India; 6 School of Veterinary Medicine, Murdoch University, Murdoch, Western Australia, Australia; 7 JH CreIndia Foundation, Guwahati, Assam, India; 8 DBT/Wellcome Trust India Alliance, Hyderabad, Telangana, India

**Keywords:** Zoonoses, Qualitative Research, Public Health, Health Services Accessibility, Community Health

## Abstract

**Introduction:**

Dog-mediated rabies disproportionately affects marginalised and geographically isolated communities, including tea-estate (TE) populations in Assam, India. Although epidemiological patterns of rabies transmission are well documented, the structural, behavioural and governance mechanisms shaping prevention and response within estate-based health systems remain poorly understood. This study used a constructivist grounded theory approach to examine how TE health systems perceive and respond to rabies risk.

**Methods:**

We conducted 26 interview events comprising 25 in-depth individual interviews and one group interview, involving 28 participants from estate healthcare, welfare, management and labour representative roles across 10 TE in Udalguri district, Assam. Purposive maximum-variation sampling ensured representation across roles, responsibilities and decision-making influence. Initial line-by-line coding, constant comparison, focused category development and memo writing informed the development of an emergent explanatory model, which subsequently informed an applied theory of change. Rigour was ensured through reflexive team discussions, consensus-based resolution of coding discrepancies and adherence to Consolidated Criteria for Reporting Qualitative Research (COREQ) criteria.

**Results:**

Responses to rabies risk were shaped by five interacting mechanisms: structural inequities in access to postexposure prophylaxis; incident-driven vigilance; fragmented governance and the absence of institutionalised dog-population management; limited health literacy resulting from ineffective communication approaches and weak surveillance systems. Collectively, these processes produced a system that remains predominantly reactive rather than preventive, perpetuating cycles of delayed care, incomplete prophylaxis and unmanaged dog-human interfaces. The emergent theory of change highlights feasible opportunities for shifting estate-based systems towards proactive rabies prevention.

**Conclusion:**

This study offers an analytically transferable health-systems model that may inform structurally peripheral or semi-autonomous labour communities across comparable plantation, industrial and labour settlements in South and Southeast Asia, identifying implementation pathways aligned with India’s National Action Plan for Rabies Elimination 2030 and the global ‘Zero by 30’ agenda.

**WHAT IS ALREADY KNOWN ON THIS TOPIC**

- Rabies prevention and control among vulnerable populations served by semi-autonomous or employer-run health systems face delayed and fragmented response to rabies risk often flawed by inherent inequities.

**WHAT THIS STUDY ADDS**

- Structural inequities, fragmented governance and weak surveillance interact to keep rabies prevention in tea-estate and similar health systems predominantly reactive rather than preventive, and proposes a systems-based theory of change to identify feasible pathways for sustained rabies control in similar semi-autonomous labour settings.

**HOW THIS STUDY MIGHT AFFECT RESEARCH, PRACTICE OR POLICY**

- By articulating a systems-based theory of change, the study identifies actionable entry points to shift rabies control from reactive to preventive approaches in semi-autonomous labour settings, with implications for policy integration, One Health coordination and future implementation research.

## Background

 Rabies is a neglected but entirely preventable zoonotic disease and remains a significant cause of human mortality globally.^1^ Approximately 59 000 people die of rabies each year worldwide, with most deaths occurring in Asia and Africa, especially among children and marginalised communities.^2^ Historically, nearly one-third of global human mortality has been accounted for by India, although a recent modelling suggests a lower but still significant human mortality of around 5726 (95% uncertainty interval 3967 to 7350).^3,4^ These statistics underscore rabies as a critical public health challenge necessitating a multisectoral coordinated One Health approach integrating human, animal and environmental health strategies. Global efforts are aligned with the ‘Zero by 30’ strategy led by WHO, Food and Agriculture Organization of the United Nations and World Organisation for Animal Health, which calls for the elimination of dog-mediated human rabies deaths by 2030.^5^

In India, the National Rabies Control Programme introduced the National Action Plan for Rabies Elimination (NAPRE 2030) in 2021, advocating convergence across the medical, veterinary and municipal sectors.^6,7^ The plan emphasises improving dog vaccination coverage, promoting responsible dog ownership and enhancing accessibility to postexposure prophylaxis (PEP), implemented through coordinated multisectoral efforts. However, despite progressive policy frameworks and improving infrastructure, progress remains inconsistent. Rural and remote areas continue to experience intermittent shortages of vaccines and immunoglobulins, and along with low coverage of dog vaccination and sterilisation rates, allows rampant viral transmission in human habitats.^8^ Experience elsewhere demonstrates the feasibility of local rabies elimination using integrated One Health models.^9,10^ For a country as large and socio-culturally diverse as India, however, scaling such models nationally remains challenging due to systemic gaps in surveillance, supply chains and governance.

The state of Assam in northeast India is one of the world’s major tea-producing regions, with numerous tea estates (TE) spread across the state.^11,12^ The TE communities reside in large, geographically contained settlements with integrated housing, schools, health facilities and workplaces within the estate environment forming a distinct occupational microsystem.^13,14^ The tea plantation belts of Assam are characterised by high population density of both humans and free-roaming dogs (FRD), with close human-animal interactions that heighten vulnerability to rabies.^13^ Historically, estate-run health systems operate parallel to the public health network, often with limited resources, oversight and workforce capacity with gaps in preventive services such as dog vaccination campaigns and in timely management of bite exposures.^15,16^

Addressing dog-mediated rabies in such complex settings necessitates understanding the social, cultural and systemic factors shaping prevention and response. While most rabies research in India has focused on epidemiological patterns or knowledge-attitude-practice surveys, the gaps in awareness, PEP uptake, surveillance and coordination persist, especially in vulnerable communities.^17^ Furthermore, most needs-assessment surveys project the community perspective, overlooking the crucial role of management in shaping access and quality of health services for workers. A prior quantitative study in selected TEs documented high dog-bite incidence and low PEP completion rates, but could not capture the contextual barriers influencing these outcomes.^13^ Furthermore, there are few explanatory qualitative studies of rabies risks and response in plantation-based or industrial-based health systems in India or Southeast Asian region. Therefore, this study adopts an exploratory qualitative approach, using constructivist grounded theory (CGT), to examine how healthcare providers and estate management perceive and respond to rabies risk, prevention and management across individual, facility and system levels. This approach enables an in-depth understanding of the processes and conditions that give rise to reactive rather than preventive responses, highlighting the need for context-specific, integrated strategies to strengthen rabies control in TE settings. Importantly, this study is also informed by health-systems and implementation-science perspectives, which emphasise that service access, governance arrangements, institutional accountability and local delivery systems shape whether effective interventions reach marginalised populations.

## Materials and methods

### Study area

This study was conducted in the state of Assam, in TEs of Udalguri district located between 26.7452°N and 92.0962°E, and sharing borders with Bhutan and the state of Arunachal Pradesh (figure 1). As a corollary to the previous retrospective study, 10 of the 22 TEs in the district were included based on administrative approval and participant consent.^13^ Prospective participants who provided informed oral consent were invited for the interviews and their details were subsequently de-identified.

**Figure 1:**
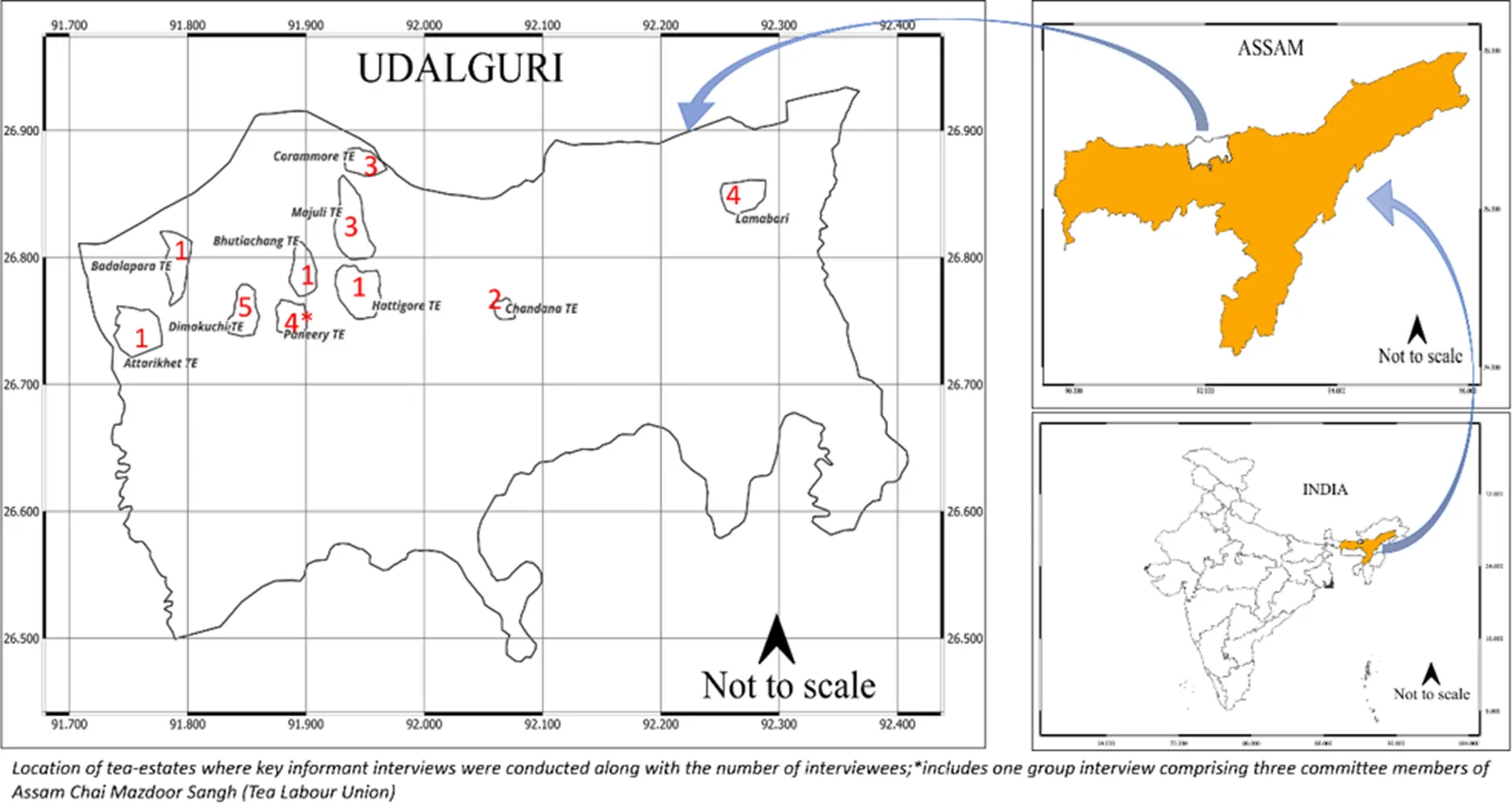
The location map of the tea estates in Udalguri district, Assam, India, where key informant interviews were conducted.

### Study design

An exploratory qualitative research design was employed, using in-depth individual interviews as the primary data collection method, supplemented by one group interview. In total, 26 interview events were conducted, comprising 25 individual interviews and one group interview involving three labour-union committee members. The group interview was conducted at one TE because of limited staff availability and time constraints. This study adopted a CGT approach. Data collection was conducted across ten TEs during May–June 2025 (figure 1).

### Sampling and data collection

Purposive maximum-variation sampling was used to include stakeholders with different roles, responsibilities and levels of influence over worker health and welfare within the TE system. Participants included medical officers (MOs), community health officers (CHOs), health assistants (HAs), welfare officers (WOs), pharmacists, deputy managers and labour union committee members. TE workers were not included because this study specifically sought to understand the perspectives of healthcare providers, welfare personnel and management staff who shape institutional responses, referral practices, awareness activities and access to PEP within estate health systems.

The interviewers’ credentials, occupational roles and qualitative-research training were documented within the research team. Participants were informed of the researchers’ institutional affiliations, roles and the purpose of the study. No non-participants were present during the interviews and no invited participants refused or withdrew. Interviews were conducted within estate health facilities or management offices in a confidential and private comfortable setting. A semi-structured interview guide was used to ensure coverage of broad domains related to dog-bite circumstances, risk interfaces, community responses, dog-population management, awareness activities and perceived strategies for rabies prevention (online supplemental file 1). The guide was developed from the study objectives and refined through team discussion and mock interviews. Although the guide was initially organised around broad domains, it was used as a flexible sequence of open-ended prompts so that interviewers could follow the natural flow of conversation and probe emerging concepts during interviews.

Senior researchers (HT, DB) assessed the face validity of the guide to ensure clarity, relevance and appropriateness. One researcher (SPK), who is conversant with the local context and has longstanding experience with TE communities, provided linguistic and cultural interpretation support. This insider positionality facilitated rapport-building, culturally sensitive interviewing and enriched contextual interpretation. The multidisciplinary composition of the research team contributed to reflexivity throughout the study, as SK’s contextual familiarity was complemented by researchers from different disciplinary backgrounds (HT, DB and RS), who critically examined emerging interpretations, challenged assumptions, considered alternative interpretations and helped minimise undue influence of individual researcher perspectives and allowed refined conceptual categories to emerge. Interviewers were encouraged to adapt the sequence and depth of questioning according to participants’ responses while avoiding leading questions and remaining attentive to their own assumptions.

All interviews were conducted in mixed Assamese and Hindi by researchers trained in qualitative data collection (SPK, RS, AUK and CS). Interviews were conducted in private settings, with confidentiality assured to encourage open discussion. Participants were encouraged to express their views freely and were informed that there were no right or wrong responses. None of the researchers held supervisory, managerial or employment responsibilities over the participants. The interviews were audio-recorded and transcribed verbatim. Field notes were taken by the interviewers during the interviews that were planned for 35–40 min while the actual duration ranged from approximately 22 to 46 min. Data collection continued until meaning saturation was achieved, whereby subsequent interviews yielded no substantially new concepts or explanatory insights relevant to the developing conceptual categories.

### Data analysis

The analytical approach was informed by CGT, particularly Charmaz’s emphasis on co-constructed meanings, constant comparison, memo writing and progressive category development.^18,19^ Qualitative data analysis was conducted manually on Microsoft Excel by two co-authors (DB, SPK) following repeated familiarisation with the data. This approach helped retain contextual meanings and address linguistic nuances through discussion with researchers familiar with the original interview languages while also strengthening reflexivity and supporting emergent theorising. Initial coding was undertaken line-by-line to identify actions, processes and meanings within the data. Through constant comparison across participants, stakeholder groups and TEs, these initial codes were progressively synthesised into focused conceptual categories. Emerging categories were continually compared with data from subsequent interviews, allowing concepts to be refined, merged or expanded as analysis progressed. Memo writing and iterative discussions among members of the research team supported further category refinement and the development of the core explanatory construct describing rabies prevention as predominantly reactive rather than preventive. This construct formed the basis of an explanatory model illustrating how rabies exposure and response unfold within the TE ecosystem, reflecting participants’ interpretations of causal pathways, constraints and enabling conditions across human and animal health domains. Ongoing comparison of successive interviews enabled the research team to judge when additional interviews were no longer contributing substantively new conceptual insights, indicating that meaning saturation had been achieved.

Following development of the explanatory model, the research team synthesised the emergent categories into a theory of change (TOC) as an applied implementation framework for identifying potential intervention pathways. Backward-mapping logic was used to link explanatory categories, including referral processes, awareness patterns, governance gaps and managerial knowledge, to relevant inputs, activities, outputs and outcomes. This process articulated connections between everyday practices and system-level goals. Iterative discussions among researchers (HT, DB, SPK and RS) were conducted throughout the analytical process to compare interpretations, refine conceptual categories and preserve participants’ intended meanings and strengthen reflexivity. Differences in interpretation were resolved through discussion until consensus was achieved.

To enhance transparency, qualitative descriptors such as ‘a few’, ‘several’, ‘many’ and ‘most’ were used when reporting the findings. These terms indicated the breadth and recurrence of perspectives across participants, stakeholder groups and TEs rather than precise numerical frequencies. They were used to convey conceptual patterns identified through constant comparison rather than to quantify participant responses.

Reporting of this study followed the Consolidated Criteria for Reporting Qualitative Research (COREQ). A completed COREQ checklist has been provided as online supplemental file 2. Credibility was strengthened through reflexive discussions and consensus-based interpretation within the multidisciplinary research team. Transferability was supported by providing contextual descriptions of the study setting, participants and TE health system. Dependability was supported through an audit trail of coding decisions, memos and iterative category development.

## Results

A total of 26 interview events were conducted, comprising 25 individual in-depth interviews and one group interview. These involved 28 participants: 25 who participated in individual interviews and three labour-union committee members who participated in the group interview. Participants represented TEs that varied in geographical settings, workforce sizes and connectivity to urban centres and hospitals. Permanent workers are directly employed by TEs and are generally entitled to employer-supported healthcare and welfare benefits, whereas temporary workers are engaged on seasonal or casual contracts and often have more limited access to these benefits. Table 1 summarises the demographic, educational and professional characteristics of all 28 participants to illustrate the diversity of stakeholder perspectives represented in the study; these data were not used for statistical comparison or inference.

**Table 1:** Characteristics of all individual participants included in the study (n=28), comprising 25 participants interviewed individually and three labour-union committee members who participated in one group interview, Udalguri district, Assam, India, May–June 2025

Characteristic	Category	N (%)
Gender	Female	9 (32)
	Male	19 (68)
Educational domain	Health sciences	17 (61)
	Human resource management/Management	8 (29)
	Humanities	3 (11)
Designation/Role in the estate system	Community health officer	5 (18)
	Medical officer	7 (25)
	Health assistant	4 (14)
	Welfare officer	4 (14)
	Manager/Deputy manager	4 (14)
	Hospital pharmacist	1 (4)
	Labour union committee members	3 (11)

* Categories within each section are mutually exclusive. Educational domain and designation are presented as separate descriptive variables. Percentages may not total 100 because of rounding. Percentages are presented solely to describe participant characteristics and sampling diversity; no statistical comparisons or inferential analyses were undertaken.

Analysis generated five inter-related conceptual categories that collectively explained the central process through which rabies prevention within TE health systems remained predominantly reactive rather than preventive. Rather than representing independent themes, these categories interacted to form the core explanatory construct and the emergent explanatory model.

### Contextual categories

#### Risk interfaces and exposure patterns

A consistent pattern of bites largely located on the legs, particularly on the thighs or below the knees was identified. The bites typically occurred in the afternoon or early evening hours, and occasionally at night when workers returned from the gardens or factories. Children and women emerged as distinct vulnerable groups. Children were commonly bitten on the hands while playing around the residential lines, and accidentally stepping on sleeping dogs, whereas women faced increased risk of being bitten while plucking leaves in the garden or during waste disposal activities.

Interviewees also noted that bite incidents were commonly reported around waste disposal dumps, markets and meat shops as these sites facilitated the congregation of FRDs. In some estates where dog-feeding practices existed, bites were also reported around these feeding points. As one WO explained, “Cases are more near the market area… dogs are always near restaurants and meat shops”. Welfare officers indicated that estates with uncontrolled dumping of waste or roadside markets experienced higher dog activity, whereas those with regulated waste disposal reported fewer incidents. Seasonal variations were mentioned by a WO highlighted periodic spikes: “June… July… August, many unprovoked bites take place, also during September and October”.

#### Pet and free-roaming dog dynamics

Participants across estates reported that some TE residents adopted FRDs (commonly referred to as stray dogs), but there was negligible awareness regarding responsible ownership. As one deputy manager noted, “Once you have a pet, you should vaccinate, but they (workers) are not doing that”. A clear distinction emerged between dogs owned by managerial staff and those adopted by workers: the former were routinely vaccinated, whereas the latter were not.

Adopted dogs were also rarely restricted, and welfare officers described blurred boundaries between pet and FRD populations, which increased overall exposure risk. A WO explained, “Our people keep dogs but don’t vaccinate, and they think home dogs won’t bite”.

In contrast, dogs showing unprovoked aggression were thought to be rabid, and participants reported that such dogs were often killed by community members as an immediate protective measure in the absence of formal animal-control mechanisms.

#### Beliefs, behaviour and complacency

Managerial staff linked poor or delayed responses to dog bites to low literacy levels, poor awareness and knowledge gaps concerning postbite wound management. Confirming this, a CHO stated, “People (workers) are mostly illiterate… they are unaware how to manage dog bites”. Several participants also shared personal anecdotes of past bite incidents, vaccinations or delayed care arising from underestimation of risk.

Participants across multiple TEs were nearly unanimous that faith healing or home remedies were now uncommon but complacency was reported to persist among some workers who perceived dog bites as ‘minor injuries’. One interviewee illustrated this with a personal example: “Earlier I was bitten but took injection only after two days… it (bite injury) looked small”. Overall, interviews indicated that tea-garden workers were moving away from traditional healing practices and more regularly seeking medical care for dog bites. A manager noted, “*…*nowadays there is no worker family that would follow home or traditional therapy for a dog bite and they invariably approach the hospitals”. Another manager added, “*…*for dog bites, they know mantras will not work… they go to hospitals; they may still approach the traditional healer for snake bites but not for dog bites”. A committee member reinforced this shift with a personal account saying, “I was bitten 12 years ago… I took vaccine”.

Despite this predominant reliance on modern medical care, a few exceptions where the old beliefs still survive were reported. An HA, who regularly interacts with bite cases, observed, “Jhaad-phoonk (traditional healing) still happens in some places… supported by the older generation… not in the tea-estate, but in nearby villages (where workers go)”.

### Core categories

#### Structural inequities in access and systemic dependence on external care

Estate health systems provided first aid but lacked the capacity to administer antirabies vaccine or immunoglobulin, creating a systemic reliance on civil and district hospitals. As one MO explained, “ARV is not available here… (we do) first aid, antibiotics, dressing, and then refer”. Most facilities therefore referred patients to district hospitals, and this referral-based structure produced marked access inequities. Permanent workers often received subsidised support, while temporary workers faced out-of-pocket payments, delayed initiation or incomplete prophylaxis. Even when vaccines were available at estate facilities, inequities persisted, as one CHO noted: “We do not give rabies injection to temporary workers… permanent workers get it free”.

Participants also described long travel distances for bite victims, inconsistent vaccine stocks and uncertainty regarding dose completion as routine barriers. Although a few managers highlighted that government hospitals provide reliable, low-cost treatment, saying, “*…*treatment is free in government hospitals… only ₹10 registration… vaccine is free”, yet they also lamented inconsistent supplies and the resulting financial burden on estates and workers: “Company bears the cost (vaccine) if government supply is not there”.

These fragmented pathways illustrate a structural dependence on external biomedical systems that undermines timely PEP access and reinforces conditional care-seeking behaviours shaped by employment status.

#### Incident-driven vigilance and the social memory of risk

Most participants described a cyclical pattern in which vigilance and adherence intensified immediately after sentinel events, almost always a rabies death, which are often accompanied by increased awareness programmes and training sessions. The intensity of such activities gradually recedes once the perceived threat wanes. Awareness sessions were rapidly scaled up after incidents, leading to temporary improvements in wound washing, early reporting and referral practices. These training sessions were conducted monthly or quarterly in outpatient departments (OPDs), schools and estate lines, typically co-facilitated by welfare and medical teams, and were triggered by bite incidents rather than offered proactively. As one CHO recalled, “(after a death case) … we did awareness in the affected colony, now we do quarterly or half yearly”.

A similar pattern was observed in reporting behaviour. A MO recounted, “last year… a child died of rabies after a scratch (from a dog) … now they even come for cat scratch”. Such incidents reshaped community behaviour, with fear motivating the early reporting. Yet these gains were reportedly short-lived, as health workers emphasised that communities ‘forget’ and revert to older ‘habits’ or draw on persistent myths once the immediacy of risk fades. This pattern reflects a crisis-triggered learning process in which extreme events temporarily recalibrate behaviour, but without reinforcement, community practices revert to as they were. These episodic shifts highlight the reactive, incident-driven nature of rabies awareness and prevention in estate settings.

MOs noted that incident-triggered initiatives continued with some consistency among health workers through integration into occupational health and safety (OHS) trainings. A WO explained, “In our (estate’s) OHS trainings, we integrate dog-bite awareness with snake bite… diarrhoea… trainings are monthly or quarterly… MOs conduct the training and (trainings) are stronger than government campaigns”. An HA added, “We do awareness monthly… in the OPD… also in lines… sometimes during the lunch break (of workers) in the garden”. These examples show that although some structured efforts exist, they remain largely reactive and reinforced by past incidents rather than ongoing preventive strategy.

#### Fragmented governance and the absence of structured dog-population control

Participants consistently highlighted the high population of FRDs as a major concern. A senior MO remarked, “Stray dogs are plenty, they come with the people (workers) even in the OPD”. The absence of any coordinated mechanism for managing FRDs across estates was a common sentiment. While periodic sterilisation was perceived as a practical solution, healthcare providers noted that no operational animal birth control (ABC) programme existed. As one HA stated, “Sterilisation (of free-roaming dogs) has to be followed”. In the absence of structured mass dog vaccination (MDV) or ABC interventions, and with no municipal coordination, occasionally individual staff arranged sterilisation through personal contacts. A deputy manager reflected this ad hoc response: “No mass vaccination and sterilisation (ever) done… I took two dogs for surgery to Guwahati earlier”.

Some participants suggested involving external stakeholders to fill this structural gap. One WO proposed non-governmental organisation (NGO)-government collaboration as a possible pathway to organised intervention, noting, “NGOs need to intervene… sterilisation and vaccination of dogs are needed… (but) under government control”.

Responsibility for dog-population control was widely perceived as diffuse, falling somewhere between estates, veterinary authorities, municipal bodies and external NGOs. Some healthcare providers favoured establishing shelters for FRDs, both to restrict movement and to facilitate adoption. A community health worker suggested, “If we have dog shelters, people may adopt the dogs”. Others questioned the feasibility of such shelters in estate settings. As one manager noted, “Dog shelters will sure help… but government has to take initiative (as it is) not possible to be taken up by estates alone”. A more sceptical view came from an MO who prioritised worker needs: “No dog shelter homes (should be) in the lines… better provide accommodations for humans (workers)”.

This governance fragmentation and decision ambiguity perpetuate a structural gap in upstream rabies prevention, reinforcing reliance on downstream, incident-driven responses instead of sustained population-level control. Estates continue to react to day-to-day risks but lack the legal mandate or structural authority to intervene meaningfully. In the absence of coordinated One Health action, reliance on short-term, estate-led measures persists, limiting opportunities for long-term prevention.

#### Preference for clear, visual and locally relevant communication approaches

Participants emphasised improving awareness about rabies, postbite wound management and PEP as key preventive measures. However, they linked the limited effectiveness of awareness activities to literacy gaps among workers. As one deputy manager observed, “Awareness… definitely, because our people (workers) in tea gardens are less educated”. Another manager noted, “Last year we did awareness after a death, and it helps the workers because they are mostly illiterates”.

Behavioural barriers also influenced uptake of instructions on wound washing after bites. A health worker highlighted the limitations of purely verbal communication: “People (workers) don’t follow (oral) instructions… most of them are uneducated… simple instructions don’t work properly”. Another HA reinforced this need, stating, “They need visual or simplified IEC”

Interviews consistently showed that workers understand messages better when health teams use clear visual tools rather than long verbal explanations. Participants reported that pictures, skits and simple demonstrations in local languages improve recall and help overcome literacy barriers. A health worker explained, “Visual media… skits or pictures in local languages would work better”.

Participants across multiple TEs pointed out the importance of child-centred information, education and communication (IEC), since children are commonly bitten during play or accidental contact. The HA expressed the need for better training and resources to deliver effective communication, as routine awareness talks alone often fail to change behaviour. One HA emphasised the value of practical demonstrations, stating, “Show the stepwise wound-wash 15 minutes with soap… then three doses… repeatedly show in OHS trainings”*.*

#### Knowledge gaps, cultural beliefs and weak surveillance

Participants generally agreed on an improvement of rabies awareness often drawing on historical recall of death incidents. Another HA noted, “…20 years ago a boy had died after a scratch by a dog… but now they report scratch (exposure) as well”. However, health officers also highlighted persistent knowledge gaps among residents regarding vaccination schedules, understanding dog behaviour and appropriate wound management. They reported that while traditional healing rituals (mantras or talismans) continued for snakebites, they were seldom used for rabies, indicating a gradual shift towards biomedical understanding.

Despite these improvements, weak surveillance mechanisms were identified as a major impediment to effective rabies control. Participants explained that documentation relied almost entirely on paper registers, with minimal follow-up and no digital reporting systems. As one MO lamented, “Registers are maintained… no computers… follow-up of cases is difficult”. TEs lacked mechanisms for tracking bite outcomes, linking data with public-sector systems, or monitoring vaccine completion. This often resulted in under-reporting. A committee member from a local tea-garden workers’ union shared, “Two rabies deaths happened in our area… not in the estate but nearby, but people (workers) are not aware”.

The data generated an emergent explanatory model (figure 2) depicting the risk posed by dog-mediated rabies perceived and managed within the TE ecosystem. The categorisation of above-mentioned conceptual constructs into gaps, barriers and enablers/opportunities provided an analytic bridge to develop a TOC. By organising the selective codes in this way, a clear pattern of relationships is visible between the conditions that generate risk, the system features that shape responses and the actions communities and providers take. Collectively, they indicate a central construct of ‘reactive not preventive’ response, underpinning the influences of recent events rather than structural planning. The enabling actions and opportunities identified comprise a series of actions, which are presented in figure 3.

**Figure 2:**
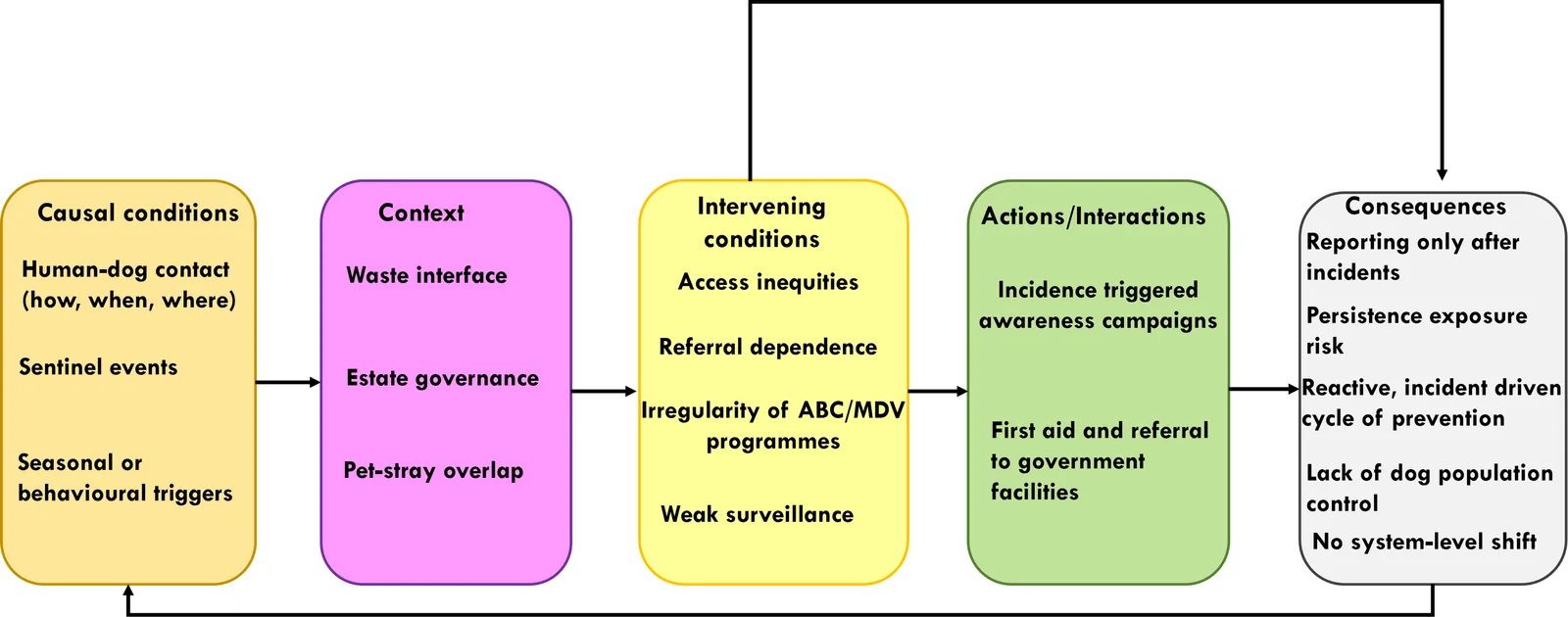
The emergent explanatory model depicting the cycle of prevailing situations, causal conditions and consequences that sustain high incidence of dog bites and dog-mediated rabies in tea estates in Udalguri district, Assam, India. ABC, animal birth control; MDV, mass dog vaccination.

**Figure 3:**
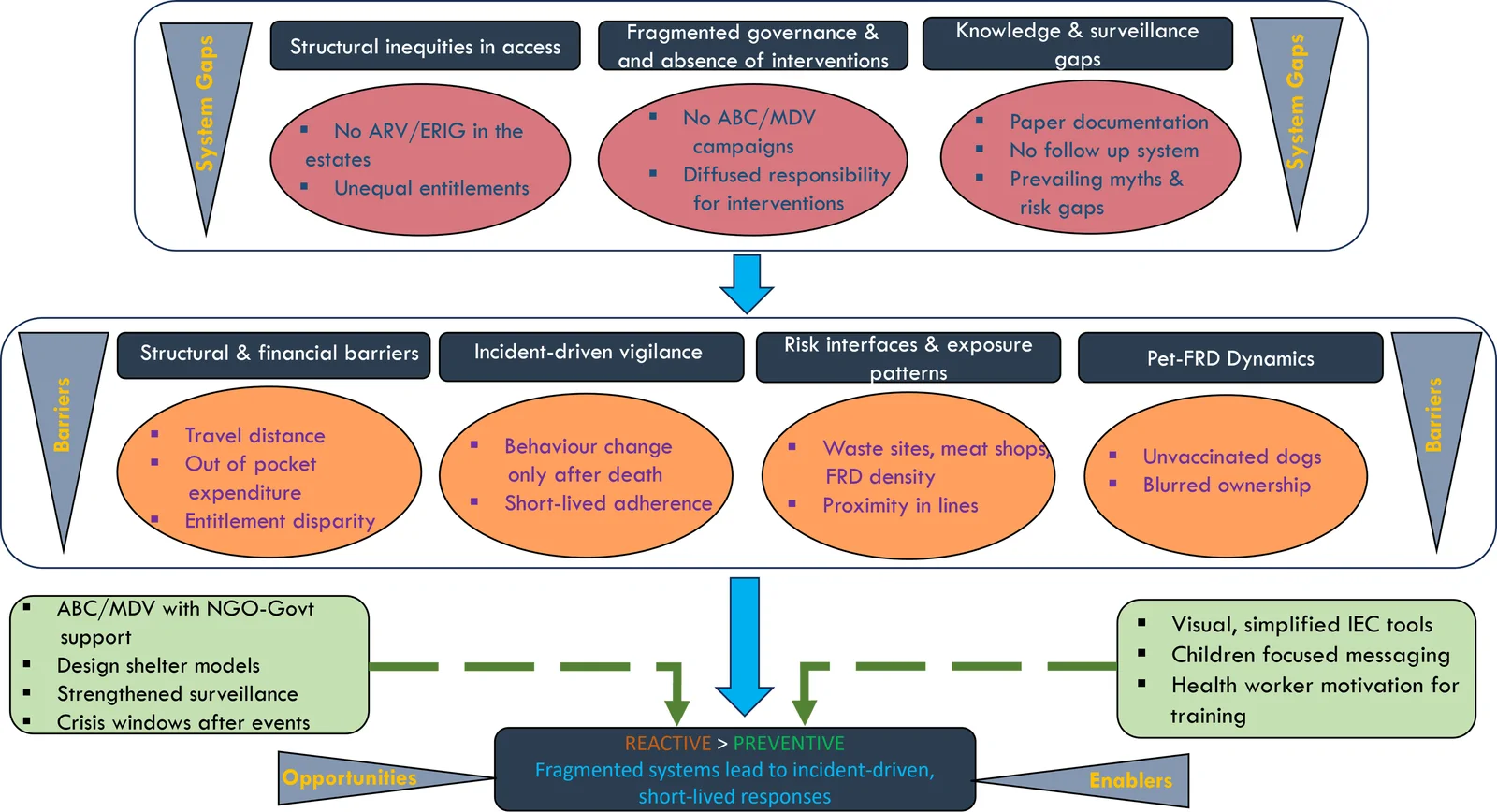
An emergent model depicting system gaps and barriers that sustain reactive behaviour, along with opportunities and enablers that shape the system towards a preventive approach to controlling dog bites and preventing dog-mediated rabies in tea-estate health systems, developed from the study in Udalguri district, Assam, India. ARV, antirabies vaccine; ERIG, equine rabies immunoglobulin; FRD, free-roaming dogs; IEC, information, education and communication; MDV, mass dog vaccination; NGO, non-governmental organisation.

### Theory of change

The TOC was developed as an applied framework following the CGT analysis, rather than as the primary grounded theory output. It translated the emergent explanatory construct of ‘reactive rather than preventive’ into potential intervention pathways for strengthening rabies prevention in TE health systems. The TOC helped to identify underlying pathways, which could realistically shift rabies risk and response dynamics in these communities. The TOC was developed by synthesising three interacting domains that were revealed through the CGT analysis. The first domain comprises prevailing contextual conditions that generate the risk of rabies transmission, characterised by high FRD population, absence of institutionalised efforts towards ABC or MDV, and external dependence for PEP. The second domain reflects systemic features that force reactive responses, such as routine general awareness or demonstrative training sessions and inconsistent referral pathways. The third domain captures the initiatives and behaviours of individuals, communities and estate health providers, which include inadequate wound washings, provision of only first-aid at the TE health facilities, postincident awareness campaigns and killing of suspected rabid FRD. These domains establish that current behaviours are driven primarily by recent incidents without active institutional planning or enhancing structural capacities. Although presented sequentially for clarity, the TOC represents an interconnected system in which several inputs, activities and outcomes influence one another simultaneously rather than through a strictly linear pathway. The TOC developed from the analysis is presented in table 2. The relationships between multiple inputs are recursive and mutually reinforcing.

**Table 2:** Theory of Change illustrating the causal pathways, inputs, activities and outcomes required to strengthen community and health-system capacities towards achieving freedom from dog-mediated rabies in TE communities*

Causal influences	Inputs	Activities	Immediate outputs	Short-term to medium-term outputs	Long-term impact
High free-roaming dog population	Promote responsible ownership, provision of shelters	Owned dog registration, government-aided and community-aided dog shelters, and identification of hotspots of dog congregation	Increased dog registration, reduced roaming and congregation of dogs, reduced dog-human conflicts	Reduced incidents of dog bites	**Freedom from dog-mediated rabies**
Absence of veterinary facilities for ABC and MDV	Technical/Resource support for ABC and MDV (mobile veterinary units)	Periodic ABC and MDV drives ensuring enhanced coverage	Reduction in free-roaming dog population, adequate MDV coverage	Decreased dog-to-dog transmission	
Inconsistent supply of PEP	Reliable PEP supply chain	Improve availability of PEP, stock forecasting tools	Consistent PEP availability and timely initiation of wound washing and PEP	High PEP completion rates	
External dependence for PEP	Training sessions for health workforce on technical and improved record keeping	Clear referral and transport protocols, training sessions for provider capacity building; digitalisation of bite registers and linkage with district surveillance	Strengthened local bite-management, reduced referrals, enhanced outbreak preparedness	Improved surveillance, decision-making and data visibility, consistent case documentation	
Knowledge gaps	Targeted IEC and demonstrative training sessions	Develop children-centric IEC, demonstrative training sessions for providers, community-level misinformation correction	Improved provider awareness and adherence to PEP protocols, responsible dog ownership	Knowledge gaps bridged	
Poor community engagement	Involvement of key opinion leaders and social mobilisers	Include the topic of dog bites, rabies in religious functions with involvement of church and temple priests, community elders and social gatekeepers, constitute dog-bite prevention committees	Strengthened collective responsibility for prevention, management and reporting of dog bites	Empowered community for rabies prevention with institutional linkages	

* The ToC components were derived from the CGT categories presented in the results, including structural inequities in access to PEP, incident-driven vigilance, fragmented governance, communication gaps and weak surveillance.

ABC, animal birth control; CGT, constructivist grounded theory; ICE, information, education and communication; MDV, mass dog vaccination; PEP, postexposure prophylaxis; TE, tea estate; TOC, theory of change.

## Discussion

This study provides grounded, contextually rich insights into how responses to dog bites are shaped within TE health systems in Assam, India. Although estate health and management staff generally recognised the severity of rabies, prevention remained predominantly reactive because of structural inequities, fragmented governance and limited access to timely PEP. The explanatory model illustrates how everyday exposure conditions, gaps in dog-population management, employment-linked entitlements and episodic community awareness collectively sustain a reactive rather than preventive system.^20^ Comparable challenges have been reported in other marginalised and semi-autonomous labour communities across low- and middle-income countries (LMICs), suggesting potential analytical transferability to settings characterised by parallel health systems and weak One Health coordination.^21^

The persistence of structural inequities for access to PEP is a key finding. Our data suggest that access to timely PEP is uneven and contingent on employment status, as only permanent workers are entitled to subsidised support, as provided in TE management policies. Furthermore, while the estates provide first-aid and counselling, they remain fully dependent on government facilities for vaccines, creating a bottleneck that disproportionately affects those with fewer entitlements. These patterns mirror evidence from other LMICs, where the availability of PEP does not translate into equitable protection; distance, transport costs, opportunity losses and precarious employment determine completion of vaccination schedules.^17,22^ Studies from other LMICs report similar vulnerabilities, with informal or low-wage workers disproportionately experiencing treatment delays and drop-outs from PEP regimens.^23–26^ The TE experience thus reinforces that rabies control cannot be dissociated from deeper questions of inequity, poverty and employment security. This dimension is acknowledged in the Indian NAPRE but not yet operationalised for private or semi-autonomous health systems as the one examined in this study.^6^

A second cross-cutting mechanism identified in this study is the predominance of incident-driven vigilance, in which rabies awareness and first-aid messaging surge immediately after a bite incident or death but rapidly diminish once the perceived threat recedes. This reactive learning dynamic is similar to observations from other LMIC settings, where communities acknowledge rabies as fatal yet delay seeking care due to risk normalisation, habituated practices or the absence of consistent reinforcement.^27^ Studies from Bhutan, Uganda and the Philippines similarly describe how sentinel deaths temporarily heighten vigilance, only for these gains to dissipate without ongoing community engagement.^28–30^ Together, these findings highlight the limitations of event-triggered communication and the need for structured, continuous behavioural reinforcement if sustained preventive practices are to be established.

Fragmented governance concerning rabies control emerged as a structural determinant shaping rabies prevention in TE settings. Although most participants acknowledged the importance of sterilisation and MDV, currently no ABC programme is being implemented through coordinated engagement with municipal or veterinary departments. In this regard, TE management bears many of the consequences of dog bites but has limited capacity to contribute to population-level dog management because it lacks the mandate, financial resources and statutory authority to implement such interventions. Limited dog-population management measures and the absence of MDV drive constrained progress on upstream drivers of rabies transmission. Estate management also expressed uncertainty regarding the feasibility of dog shelters and the resources required to maintain them. These gaps stand in contrast to One Health principles and the global ‘Zero by 30’ framework, which position MDV and intersectoral coordination as central to elimination efforts.^31^ This study also highlights communication challenges that limit behaviour change among vulnerable groups. Although awareness sessions are reportedly common, their impact is limited because of the use of didactic methods instead of participatory approaches, creating a current gap in health literacy among the estate population. Participants emphasised that simple visual tools, demonstrations of immediate care practices in local language and child-focused communication would be effective. This suggestion is supported by studies indicating that pictorial and school-based IEC materials can strengthen early reporting and appropriate first-aid practices in low-literacy settings.^16,32^

Weak bite-surveillance mechanisms are a critical barrier in identifying the high-risk areas and population groups resulting in gaps in budgeting, resource allocation, preventive equipment, capacity building and referral to hospitals. Our study also found that record-keeping within estates remains manual, paper-based and inconsistent,^13^ with no linkage to national digital surveillance platforms such as the Integrated Disease Surveillance Programme for human health or the National Animal Disease Reporting System (NADRS/NADRS-2.0) for animal health. This contributes to under-reporting and an absence of feedback loops, limiting the visibility of bite incidence and treatment completion.^33^ Similar gaps are widely documented across LMICs, particularly within semi-autonomous health systems serving plantation or industrial communities.^34,35^ This strengthens the urgent need for an integrated surveillance mechanism between estate-based and public health systems.

The reactive nature of rabies prevention in the estates reflects structural gaps in access, governance and surveillance, which also help explain episodic surges in awareness and the persistence of unaddressed ecological drivers of exposure. Our findings show a clear differentiation of risk and responsibility within the TE system, which directly shapes rabies-prevention practices. Field-based workers such as tea-leaf pluckers, factory workers, fertiliser applicators and daily-wage workers were perceived to face particularly high exposure because they constantly encounter FRDs during outdoor tasks. Clerical staff do not enter high-risk areas, but their families, especially children, remain vulnerable due to frequent movement across estate spaces. Managers, MOs and WOs carry the financial and operational load of responding to bite incidents in several estates. HAs, although not commonly described as occupationally exposed to dog bites, serve as the first point of contact, manage bite cases, maintain records and coordinate referrals to the primary health centre. Existing structures such as labour committees represent an important but underused platform for strengthening community engagement, awareness and follow-up of bite victims.

FRDs were consistently identified as a persistent challenge, driving recurrent bite injuries and community anxiety. These insights show that rabies prevention in TEs requires differentiated, role-specific strategies that acknowledge varying exposure levels and operational responsibilities across the workforce. The emergent model generated by this qualitative study advances understanding of rabies prevention in semi-autonomous and structurally peripheral health systems. The interactions show how causal conditions (human-dog interfaces, sentinel events) unfold within contextual constraints (governance gaps, waste interfaces) and are mediated by intervening conditions (access inequities, weak surveillance) to produce downstream actions (first aid, referral, awareness bursts) that ultimately fail to shift the system towards sustained prevention (figure 2). Interpreting these findings through health-systems and implementation-science lenses suggests that the observed gaps are not simply deficits in awareness or behaviour, but reflect structural constraints in service delivery, governance, accountability and access.

A community-based study from Sri Lanka documented high compliance with immediate wound washing and early reporting despite structural barriers, providing a useful comparison with our findings.^36^ Similarly, active participation in ABC and mass dog-vaccination campaigns in Bhutan and Kenya contrasts with the largely passive approach to dog-population control described by participants in this study.^22,37^ In other instances, such as rural Indonesia and the Philippines, locally driven dog-vaccination initiatives led by village councils or community groups demonstrate bottom-up governance structures that were not evident in participants’ accounts from Assam’s TEs.^38,39^ Finally, while participants in our study described high trust in biomedical care and minimal reliance on traditional healers, studies from Nepal, Ethiopia and parts of India have shown substantial delays in PEP due to the use of traditional healing practices.^40,41^ These contrasting findings highlight that rabies-prevention behaviours are not uniform across LMICs and underscore the decisive role of community agency in shaping the feasibility and effectiveness of rabies elimination strategies.

These recommendations represent the authors’ interpretation and translation of the empirical findings’ potential implementation strategies, rather than direct recommendations expressed uniformly by participants. Nonetheless, the findings have clear implications for policy and practice. Integrating TE representatives into district-level Zoonoses Committees and state NAPRE implementation structures could improve referral coordination, strengthen surveillance and ensure that plantation populations are not excluded from core elimination activities.^42^ Expanding ABC and mass dog-vaccination interventions through coordinated engagement between local municipal bodies, veterinary departments and estate authorities would begin to address upstream drivers of transmission, consistent with One Health principles. Implementation of these recommendations would, however, require strengthening digital infrastructure, referral systems and routine monitoring capacity within TE health services.

Although derived from TEs in one district of Assam, the model may have analytical relevance to other microsystems in rabies-endemic settings. Evidence from mining communities, brick-kiln sites, industrial enclaves, refugee settlements and informal peri-urban areas demonstrates similar patterns of institutional fragmentation and intense human-animal interfaces that heighten zoonotic risk and impede coordinated prevention efforts.^43–46^ As the model is conceptually grounded in identified gaps, barriers and enablers, it gives a unique opportunity to design an integrated, multisectoral strategy aligned with One Health principles and national rabies-elimination agendas (figure 3).

## Limitations

The study relied on self-reported practices and perceptions, which may have been subject to recall or social desirability biases, particularly regarding adherence to guidelines or estate-level responsibilities. The captured perspectives came from healthcare providers, welfare personnel, management staff and labour-union representatives. The exclusion of workers, children and community members limited insights into household-level decision-making and lived experiences. Furthermore, respondents did not routinely observe bite-management practices or delivery of information, education and communication activities; therefore, the findings reflect reported rather than directly observed behaviour. Finally, triangulation of reported bite incidence and PEP completion with district surveillance was not possible, although weak reporting system itself emerged as an operational gap.

## Conclusion

This study reveals that rabies prevention within TE health systems of Udalguri district in Assam, India, remains predominantly reactive, shaped by structural inequities, fragmented governance, weak surveillance and event-triggered episodic awareness. Strengthening preventive capacity will require integrating estates into district and state rabies-governance structures, expanding ABC and MDV programmes, improving communication approaches, ensuring equitable access to PEP and considering dog-shelter provision where appropriate and feasible. The study generated a conceptual model that may have analytical transferability to other occupational or semi-autonomous health systems with comparable organisational and contextual characteristics.

## Supplementary material

10.1136/bmjph-2025-004805 online supplemental file 1

10.1136/bmjph-2025-004805 online supplemental file 2

## Data Availability

Data are available on reasonable request. All data relevant to the study are included in the article or uploaded as supplementary information.
