# Supplementary material for: Rabies risk and response in tea-estate health systems in Assam, India: a constructivist grounded theory analysis
Source: BMJ Public Health. 2026 Sep 30;4(3):e004805. doi: 10.1136/bmjph-2025-004805 (PMC13629953; doi:10.1136/bmjph-2025-004805)
Supplement: Supplementary file 1 [file bmjph-4-3-s001.pdf]

Supplementary file 1

KII- INTERVIEW GUIDE (TE management)

1. Greetings! Could you please shed some light on the position you hold in the organisation and for how long have you been working in this position?
2. How would describe the situation of dog-bites and rabies in your estate? Has there been a death due to rabies in near past? Can you elaborate on any cases of rabies/dog-bite that captured your attention?
3. 3. What are the reasons in your opinion for dog bites in your estate? Do you think the dogs are provoked to attack? What could be the reasons of provocation? Alternatively, do you think the dogs attack unprovoked? If so what are the circumstances of such attacks?
4. According to you, what would be most common interfaces such as waste disposal sites, worker lines, meat shops, etc.) that facilitate high incidence of dog bites?
5. Which part of body do the bites mostly take place? Have you observed any specific part of the day where dog bite incidences are more (morning, afternoon, evening, night)?
6. What category of dogs would you consider are mostly responsible for the dog-bite incidences (domesticated, free roaming within the estate, free-roaming outside the estate, etc.)?
7. Do you think dog shelters would help in curbing/ bringing down the cases of dog-bites? If yes, elaborate. If no, then why not?
8. Has there been any awareness campaigns, dog-mass vaccination or sterisation campaigns in your estate? Has there been any effort to control the dog population or promote responsible dog ownership?
9. Has there been any effort from the government health sector to control the problem?
10. Do you conduct any awareness campaigns about rabies in the community?
    Are there any other activities (apart from campaigns) you undertake for community awareness on rabies?
    If yes, elaborate. If no, then why not?

11. Do you think raising community awareness on rabies will help in bringing down the cases of dog-bites?
12. How do you think we can bring down dog-bite incidences and rabies cases in tea-estates?

<u>We appreciate the time taken by you to answer these questions, this will greatly help our study. Thank you!</u>

This semi-structured interview guide was developed from the study objectives and relevant literature on dog-bite risk, rabies prevention, and tea-estate health systems. The guide was reviewed by senior researchers for clarity, relevance, and contextual appropriateness, and was refined through team discussions and mock interviews before field use.

The questions were initially organised around broad thematic domains. However, these headings were not retained in the field version so that interviewers could move flexibly between topics according to the natural flow of conversation rather than follow a rigid sequence. Thematic domains were retained during interviewer training to ensure that all core areas were covered across interviews.

The guide was used as a flexible set of open-ended prompts rather than as a structured questionnaire. Interviewers could vary the sequence and wording of questions, seek clarification, request examples, and probe emerging concepts while avoid leading questions. Not every prompt was asked in exactly the same manner during every interview. The same guide was adapted for the group interview, with participants encouraged to discuss and respond to one another's perspectives.

Common probing prompts included:

1. Could you explain that further?
2. Could you provide an example?
3. Why do you think this happens?
4. What usually happens after such an incident?
5. Who is responsible for responding to this issue?
6. What could be done differently?
