# Supplementary material for: Rabies risk and response in tea-estate health systems in Assam, India: a constructivist grounded theory analysis
Source: BMJ Public Health. 2026 Sep 30;4(3):e004805. doi: 10.1136/bmjph-2025-004805 (PMC13629953; doi:10.1136/bmjph-2025-004805)
Supplement: Supplementary file 2 [file bmjph-4-3-s002.pdf]

Original research: Rabies risk and response in tea-estate health systems in Assam, India: a constructivist grounded theory analysis

Consolidated criteria for reporting qualitative studies (COREQ): 32-item checklist

**Domain 1: Research team and reflexivity**				**Page no.**
**Personal Characteristics**				
1	Interviewer/facilitator	Which author/s conducted the interview or focus group?	SK, RGS, AUK, CS	8
2	Credentials	What were the researcher's credentials?	PhD/ Masters of Public Health as applicable	
3	Occupation	What was their occupation at the time of the study?	Principal Investigator/Post-doctoral scholar/Public Health researcher/ research scholar as applicable	-
4	Gender	Was the researcher male or female?	Three female interviewers and one male interviewer	-
5	Experience and training	What experience or training did the researcher have?	The interviewers had received training in qualitative data collection through a qualitative-research workshop and in-house training.	8
Relationship with participants				
6	Relationship established	Was a relationship established prior to study commencement?	No prior relationship existed with most participants. One researcher had longstanding familiarity with TE communities but held no supervisory, managerial, or employment relationship with participants.	7
7	Participant knowledge of the interviewer	What did the participants know about the researcher?	Participants were informed of the researchers' institutional affiliations, research roles, and the purpose of the study	8
8	Interviewer characteristics	What characteristics were reported about the interviewer/facilitator?	The manuscript reports the local and cultural familiarity of one researcher, the multidisciplinary backgrounds of the analytical team, interviewer training, and the	8

			absence of supervisory or employment relationships with participants	
Domain 2: study design				
Theoretical framework				
9	Methodological orientation and Theory	What methodological orientation was stated to underpin the study? e.g. grounded theory, discourse analysis, ethnography, phenomenology, content analysis	Constructivist Grounded Theory, informed by Charmaz.	8
Participant selection				
10	Sampling	How were participants selected?	Purposive maximum-variation sampling approach was followed	7
11	Method of approach	How were participants approached?	Prospective participants were approached in person at participating tea estates following administrative approval.	6
12	Sample size	How many participants were in the study?	28	7
13	Non-participation	How many people refused to participate or dropped out?	No invited participants refused participation or withdrew.	8
Setting				
14	Setting of data collection	Where was the data collected?	Estate health facilities and management offices.	8
15	Presence of non-participants	Was anyone else present besides the participants and researchers?	No non-participants were present	8
16	Description of sample	What are the important characteristics of the sample?	Healthcare providers, welfare personnel, management staff, and labour-union committee members; additional demographic, educational, and professional characteristics are presented in Table 1.	12
**Data collection**				
17	Interview guide	Were questions, prompts, guides provided by the authors? Was it pilot tested?	Yes. The semi-structured interview guide is provided as Supplementary File 1 and was refined through team discussion and mock interviews.	8
18	Repeat interviews	Were repeat interviews carried out? If yes, how many?	No	-

19	Audio/visual recording	Did the research use audio or visual recording to collect the data?	Audio recorded	8
20	Field notes	Were field notes made during and/or after the interview or focus group?	Yes	8
21	Duration	What was the duration of the interviews or focus group?	Approximately 22–46 minutes	8
22	Data saturation	Was data saturation discussed?	Meaning saturation was assessed through ongoing comparison of successive interviews; data collection ceased when additional interviews yielded no substantively new conceptual insights	9
23	Transcripts returned	Were transcripts returned to participants for comment and/or correction?	No	-
**Domain 3: analysis and findings**				
**Data analysis**				
24	Number of data coders	How many data coders coded the data?	Two (DB, SK)	8
25	Description of the coding tree	Did authors provide a description of the coding tree?	The progression from initial codes to focused conceptual categories, the core explanatory construct, and the explanatory model is described in Section 2.4 and illustrated in Figure 2.	10
26	Derivation of themes	Were themes identified in advance or derived from the data?	Conceptual categories were derived inductively from the interview data through initial coding, constant comparison, memo writing, and progressive category development.	9,10
27	Software	What software, if applicable, was used to manage the data?	Microsoft Excel was used to organise coded data; analysis and interpretation were conducted manually.	9
28	Participant checking	Did participants provide feedback on the findings?	No; participant checking of transcripts or findings was not undertaken.	-
**Reporting**				
29	Quotations presented	Were participant quotations presented to illustrate the themes / findings? Was each quotation identified?	Yes. Quotations were attributed by participant role or cadre, while personal identifiers were omitted.	12-21

30	Data and findings consistent	Was there consistency between the data presented and the findings?	Yes; quotations and participant accounts are presented alongside the corresponding conceptual categories.	12-21
31	Clarity of major themes	Were major themes clearly presented in the findings?	Yes; five interrelated conceptual categories and the core explanatory construct are clearly presented.	13-17
32	Clarity of minor themes	Is there a description of diverse cases or discussion of minor themes?	Yes; contextual categories, divergent perspectives, and exceptions: including differing views on dog shelters and residual traditional-healing practices are reported.	12-21
